# Fractal conceptualization of intumescent fire barriers, toward simulations of virtual morphologies

**DOI:** 10.1038/s41598-019-38515-9

**Published:** 2019-02-12

**Authors:** Gizem Okyay, Anil D. Naik, Fabienne Samyn, Maude Jimenez, Serge Bourbigot

**Affiliations:** University of Lille, CNRS, ENSCL, UMR 8207, UMET, Unité Matériaux et Transformations, F-59000 Lille, France

## Abstract

By limiting the heat spread during a fire hazard, intumescent coatings are important components of passive protection systems. They swell due to heat induced reactions of micro constituents and are transformed into carbonaceous porous-like media, known as intumescent chars. Their multiscale inner structures, key elements of performance, are costly to predict by recurrent and large scale fire testing while numerical simulations are challenging due to complex kinetics. Hence, we propose a novel approach using the fractal theory and the random nature of events to conceptualize the coating expansion. Experimental specimens were obtained from fire protective coatings exposed to bench scale hydrocarbon fire. Mass fractals were evidenced in the slices of 3D sample volumes reconstructed from X-ray microtomography. Consequently, geometrical building blocks were simulated by random walk, active walk, aggregation-like and site percolation: physical-chemical modes of action were inherent in the attribution of the randomness. It is a first demonstration to conceptualize different types of intumescent actions by a generalized approach with dimensionless parameters at multiscale, thus eliminating the simulation of complex kinetics to obtain a realistic morphology. Also, fractal results brought new evidence to former chemical analyses on fire test residues trying to explain the kinetics of expansion. Expected outcomes are to predict virtually the reaction of fire protective systems hence to speed-up the assessment of fire performance through computed properties of virtual volumes.

## Introduction

In order to assess the fire safety performance of materials, one should examine the two components: the flame itself and the materials upon burning. Those can be examined separately or together, depending on the topic and the research interests^1,2^. Studying the constraints of fire scenario and the interactions of the evolved heat with its surroundings can become cumbersome due to the complex kinetics and all modes of transfers involved^3^. Hence, one way to investigate the fire performance is to quantify physical properties, decoupled from complex kinetics if possible, and as a function of time when available. For the fire performance tests where convection and conduction dominate (e.g. furnace tests), the emphasis is given to the condensed phase, more precisely to the properties of the carbonaceous solid residues as the burning of materials leads to the combustion products such as soot, ash and char.

**Figure 1:**
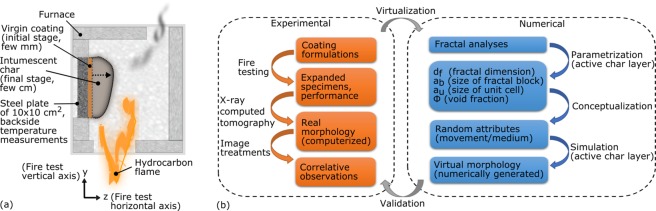
(**a**) Schematic illustration of our laboratory scale furnace testing on fire protective coatings to mimic hydrocarbon fire scenario^7,39^. The expansion pattern and the internal structure of the expanded char specimens are the key elements for fire protection besides their chemical compositions. (**b**) Overview of the methodology proposed in this study for the shape parametrization of chars and the virtual generation of their active layer.

In this study, intumescence was selected as a key element of passive fire protection where the condensed phase action dominates^4^. In general terms, intumescence is the expansion of a material when subjected to external constraints. For fire protective coatings, intumescence is due to the thermal stimulus: the polymeric coatings undergo chemical reactions coupled to physical changes when exposed to heat leading to a bubbling/expansion combined with simultaneous charring reactions producing the final intumescent char^5^. Those porous residues act as heat barrier between the flame and the underlying substrate^4^. It is a widely used concept to fire protect materials, such as steel structures^6^. As an illustrated example, our laboratory bench-scale fire testing^7^ of intumescent coatings is presented in Fig. 1a. During this fire scenario, coatings of few millimeters expand up to few centimeters; the expansion pattern and the multiscale inner morphology of charred residues play an important role in their performance.

The behavior of an intumescent specimen exposed to fire is almost impossible to fully predict a priori, due to complex correlations between the sample and the flame chemistries, combined with the thermal and mechanical actions and temperature-time dependent physical properties^5^. In this case, it is necessary to decouple, conceptualize and generalize some groups of parameters. Up-to-date data report extensive information about the chemical actions^6,8^ whereas the prediction of morphology is limited^9,10^ as the analysis and generation of representative geometries can rapidly become cumbersome due to multiple parameters (pore ratio, orientation, size, distribution, shape, etc.) related to inner morpology^9,11,12^. In parallel, researches were performed to describe the behavior of fire barriers, in steady^13,14^ or unsteady^10,15^ approaches, either numerically or experimentally to determine the effective properties. While the expansion rates were quantitatively given for unsteady approaches, the description of the inner morphology was performed only upon qualitative observations^16^, to assess possible synergies between the ingredients of coatings^17^, to determine kinetics of degradation^18^, to obtain input data for simulations with some quantitative determinations for pore size distributions^9^. It is possible to simulate the kinetics^19,20^ and to resolve fire behavior through effective medium properties in inverse^14^ or direct^9,20^ methods but it is challenging to integrate morphology information in all modeling and simulation tools. It requires important computational effort and the results are dependent on extensive experimental data providing time and temperature dependent properties^20^. On the other hand, conventional bubble models are not universal to all intumescent coatings^6,14,20^ and are not representative of novel intumescent formulations based on self-expanding systems^6^.

Better interpretation of physical measurements based on morphology are needed both for numerical or experimental modeling and simulations^7,9,12,20^, as studies evidenced that the microscale structural properties of chars are susceptible to affect their fire performance^9,12^. Recent studies^12,14^ propose a parametrization of expanded coatings regarding the apparent physical properties. For example, they determine the apparent properties by inverse computations and change the boundary conditions to tune the heat flow trough the sample; they don’t take into account morphology details which disappear by the definition of effective properties. Nevertheless the porous morphology (in addition to the material composition and expansion ratio) is susceptible to affect the performance of expanded coating either by the mass density (e.g. strength, conduction and radiation), the inner geometry (e.g. radiation through pores) or inner tortuosity (e.g. radiation or convection due to reactions or pyrolysis through pore walls). All the information can not by completely reduced to the expansion ratio nor to the effective properties of intumescent coatings, noting the considerable errors and uncertainties in the models^12,14^. To reduce prediction errors, the effective properties shall be fine-tuned by morphology parameters because the complex geometry will modify the ratio between the transfer modes and the strength of the material. Therefore, the realistic inner char structure and its multiscale properties should be examined for further consideration of both the mechanical and thermal fire performance. At present, no work exists, which would offer unique dimensionless solution for the description of coating expansion pattern: the challenge is to parametrize quantitatively the inner structure from macro scale down to micro scale and, if applicable, to propose representative virtual geometries with a universal method for different modes of action of formulations.

Thereby, we propose a novel approach to analyze and to conceptualize fire protective intumescent coatings by using fractal theory^21^. The methodology consists in a combined experimental-numerical approach as resumed by the flowchart in Fig. 1b. Fractal theory could describe the formation/fabrication of materials in nature (e.g. atmospheric fine particles and pollution, space interstellar dust, structural materials and additives such as ceramics, paints and colloids) and also many hazardous phenomena^21–25^. Fire being one of the latter, fractality has been applied to hazardous fires, the flame and smoke particles^26–28^ in order to study and model virtually their patterns, interactions or effective properties. Note that hydrocarbon combustion is a good example of the fractal parametrization: the transfer correlations (e.g. the effective properties of sooty medium) can be tuned by the fractal dimension of the particulates dispersed in the gaseous phase without simulating the complex aggregate geometries^27^. Nevertheless, the fractality of fire barriers has never been investigated in the condensed phase. The novelty lies in this step: we investigate the possible existence of an analogy between the gaseous phase (e.g. gas phase soot, aerosol, flame) and the condensed phase (e.g. solid residues, intumescent chars) in terms of the applicability of the fractal theory in hydrocarbon fire scenario.

The proposed characterization methodology was applied on two different intumescent systems: an epoxy-based coating formulation, representing conventional bubbling system, and a silicone-based coating formulation, representing novel self-expanding system^6^. These systems were selected upon their previously reported good thermal and structural performances^7,29,30^. Their standard fire testing results were discussed in reported studies among other intumescent formulations, but their morphology has never been investigated. Therefore, we will report our quantitative results on intumescent morphology and bring novel insights and implications in experimental fire testing and numerical simulations.

Specimens were collected from expanded coatings after bench scale furnace testing mimicking hydrocarbon fire (Fig. 1a). Expanded specimens, obtained from the fire testing of epoxy-based and silicone-based formulations, will be named hereafter as “epoxy char” and “silicone char” respectively. Specimens were observed by tomography and microscopy. Correlative image observations and analyses were performed at multiscale. Fractal analyses were applied on slice images of 3D volumes obtained from computed tomography. As numerical analyses indicated fractal behaviors for both formulations, an immediate application was presented for the numerical generation of intumescent morphologies: geometrical building blocks were virtually simulated by random generation algorithms. The input parameters were estimated from fractal results combined with correlative imaging and image treatments, as summarized in Fig. 1b. Finally, the comparison of tomography sections with their corresponding virtual numerical generations validated our proposed approach in this study. Proposed method allow a first demonstration of fractal parametrization intumescent morphology. Our approach was generalized to different modes of actions of fire protective coatings, thus eliminating the difficulty of simulating the kinetics to obtain a virtual representative inner geometry.

## Results and Discussions

### Experimental overview: Multiscale components

**Figure 2:**
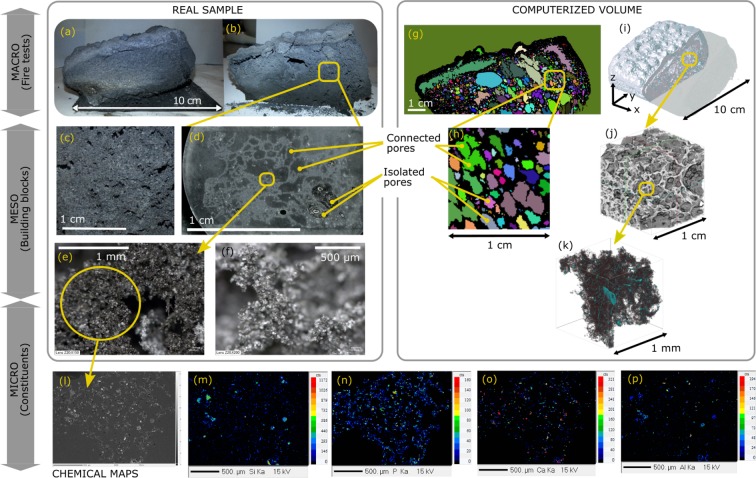
Correlative observations of the epoxy char inner structure. (**a**) Picture of the real char sample after fire test. (**b**,**c**) Pictures of the middle cross-section, cut after performing CT. More sample images are provided in Supplementary File. (**d**) Digital microscope image of char section embedded in epoxy resin. (**e**,**f**) Digital microscope images of the bare cut section. (**g**,**h**) Middle slice images from CT reconstructions: Connectivity of pores were visualized by watershed image analysis and the different colors indicate the presence of separated regions. Extended figures are in Supplementary File. (**i**) 3D reconstruction of undamaged sample from CT. Rotation movie is in Supplementary Data. (**j**) Zoomed 3D section from CT. (**k**) 3D reconstruction from HRCT on cut sample: light elements are in grayscale and the elements heavier than carbon are highlighted in color. Rotation movie is in Supplementary Data. (**l**) BSE (back scattered electrons) recording in EPMA (electron probe microanalyzer) of the resin embedded sample. (**m**–**p**) Corresponding distributions of major chemical constituents other than carbon, recordings from EPMA. Extended figures are in Supplementary File.

**Figure 3:**
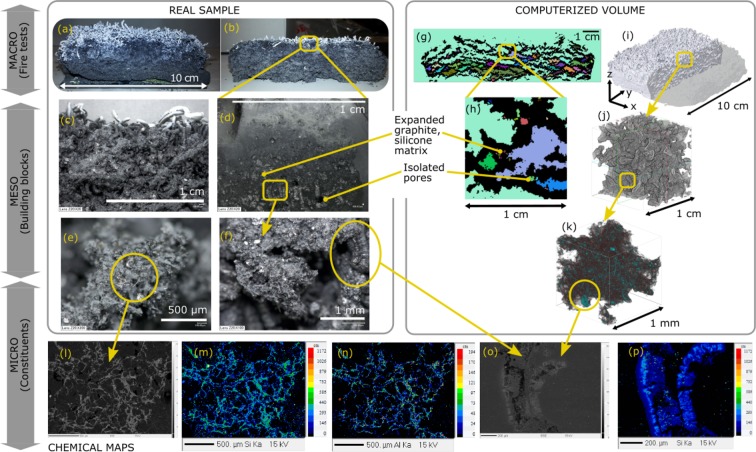
Correlative observations of the silicone char inner structure. (**a**) Picture of the real char sample after fire test. (**b**,**c**) Pictures of the middle cross-section, cut after performing CT. More sample images are provided in Supplementary File. (**d**) Digital microscope image of char section embedded in epoxy resin. (**e**,**f**) Digital microscope images of the bare cut section. (**g**,**h**) Middle slice images from CT reconstructions: Connectivity of pores were visualized by watershed image analysis and the different colors indicate the presence of separated regions. Extended figures are in Supplementary File. (**i**) 3D reconstruction of undamaged sample from CT. Rotation movie is in Supplementary Data. (**j**) Zoomed 3D section from CT. (**k**) 3D reconstruction from HRCT on cut sample: light elements are in grayscale and the elements heavier than carbon are highlighted in color. Rotation movie is in Supplementary Data. (**l**) BSE (back scattered electrons) recording in EPMA (electron probe microanalyzer) of the resin embedded sample. (**m**–**p**) Corresponding distributions of major chemical constituents other than carbon, recordings from EPMA. Extended figures are in Supplementary File.

Epoxy-based and silicone-based coatings were subjected to bench-scale hydrocarbon fire testing. The details of the coating formulations and the test conditions are given in Methods section. The results of fire testing (temperature-time curves) will be discussed in the section on conceptualization. Here, we present the experimental studies elucidating the inner structures of samples. The expanded samples collected from fire tests are illustrated in Figs 2a and 3a. X-ray computed microtomography (X-ray *μ*CT, abbreviated as CT throughout the study) was applied on intact char specimens. The CT reconstructed volumes of the undamaged chars gave a resolution of 81 *μm*/*pixel*. CT was repeated at higher resolution (abbreviated as HRCT throughout the study) on tiny samples collected thoroughly in the middle sections of the cut specimens. HRCT reconstructed volumes had a resolution of 1 *μm*/*pixel*. Multiscale computerized volumes were obtained as presented in Figs 2i,k and 3i,k, at the dimension scales “micro-meso-macro” for the condensed residues of the engineered coatings. In parallel to tomography, naked-eye and microscopy observations were performed on the real char specimens. Multiscale visual observations show the evidence of different geometrical constituents in the charred coatings issued from fire testing. At experimental (macro) scale, the sample size is generally around few centimeters for both types of coatings. However, the structure of the pores, the size of the geometrical building blocks and the microscale constituents vary between both formulations. First an expanded epoxy based coating will be examined as it represents a conventional system where the active reactants lead to continuous bubbling. Second, an expanded silicone based coating will be examined as it represents a relatively novel formulation where the reactants lead to rapid self-expansion of the coating without any foaming agents.

#### Conventional bubbling system

The epoxy based coating is a classical intumescent system, containing a carbon source, an acid source, a blowing agent for the foaming reactions^4^ and additives such as fibers to ensure mechanical strength of the char. Expansion is mainly due to blowing agents reacting under heat exposure and forming bubbles in the hot molten layer with subsequent charring reactions^30^. The geometrical building blocks are the pores (bubbles) at multiscale, as shown in Fig. 2b,d,e. According to reported data^4,6,30^, the active layer causing the expansion was presumed to be the hot molten bottom part because the top layer was rapidly charred during expansion. When the chemical reactions reach their limits and cannot produce more blowing agents, the coating is completely charred, solidified (and partly pyrolized depending on the duration of flame exposure). Major chemical constituents of the middle layer of char at microscale are presented in Fig. 2m–p. Those distributions are the resulting elements of the physical and chemical interactions between the binder, the blowing agents and the additives such as the fibers. The microscale constituents were visualized in 3D thanks to the HRCT of Fig. 2k. As observed in Fig. 2g,h, pores of different sizes follow a highly non-uniform distribution. Most of the pores on the top part of the epoxy char are interconnected (Fig. 2g), whereas isolated bubbles are observed in the bottom part as shown in Fig. 2g. It was presumed that the interconnection at the top part of the char is due to coalescence followed by the explosion of pore walls and their oxidation or pyrolysis whereas at the bottom of the char it is considered mostly due to a partial bubble coalescence^10^. The partial coalescence of bubbles was better observed in the mesoscale as presented in the optical microscopy image (Fig. 2d) compared to the analyzed CT section (Fig. 2h). This action is to be kept in mind during conceptualization in the following sections on numerical analyses and virtual generations.

#### Self-expanding system

The silicone-based formulation is a self-expanding system based on expandable graphite (EG) mixed with a silicone binder. Silicone based formulations are relatively recent and they were reported to exhibit good fire performance compared to conventional intumescent systems^6^. A two step expansion action was presumed for this system based on reported data on similar formulations^29,31^. First, when temperature increases enough to activate the chemical reactions, the rapid sublimation of sulfuric acid inserted in the graphite platelets leads to growing graphite worms (Fig. 3c) attached to a silicone binder^29^ (Fig. 3d,f). Then, with further increase of the temperature, silicone binder is presumed to partly bond chemically to the graphite platelets (Fig. 3o,p), and partly penetrate the pores; it undergoes charring and solidifies (Fig. 3l–n). Contrary to a classical system, the mechanism of action is not due to foaming agents but it is due to the expansion of the EG platelets in the silicone binder. This self-expansion action will be reminded in the following sections for the numerical conceptualization. At the end of fire testing, the silicone char contains rather uniformly distributed superposed layers as observed in Fig. 3b,c,j. The voids separating those layers are more or less interconnected as shown in Fig. 3g,h. The size distribution of the worm-like shapes is not uniform; the smallest ones reach few hundreds of microns, as observed in Fig. 3f,o. The difference between the characteristic sizes (of the silicone matrix, of the smallest graphite worms and of the fibers/additives) is clearly observed in the HRCT reconstruction of Fig. 3k.

In the following section, the results of the fractal analyses on 3D reconstructions of epoxy (Fig. 2i,k) and silicone (Fig. 3i,k) chars are presented. The above observations were taken into account while determining numerically the fractality limits and the dimensions of the geometrical building blocks of both specimens.

### Numerical Analysis: Parametrization of inner morphology

**Figure 4:**
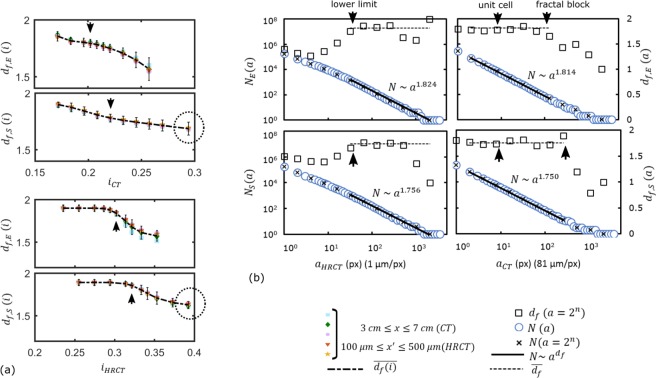
(**a**) Fractal dimension *d*_*f*_ versus binary threshold at gray level *i* (computed over the linear transition regions for both CT and HRCT data). Arrows indicate the selected *i* for the computation of the global mesoscale *d*_*f*_, matching with the *i* value given by automated thresholding. Encircled regions indicate the possible existence of second fractality of the silicone based polymeric matrix itself. (**b**) Box count and linear regression on the middle slice of tomography volumes. Arrows indicate the determined lower limit of fractality, the unit cell size of mass fractals and our upper limit of fractality at meso/macroscale. [Symbols: *N* is the counted number of boxes, *a* is the size of boxes in pixel, *d*_*f*_ is the fractal dimension, *x* is the coordinate of CT slices, *x*1′ is the coordinate of HRCT slices, *i* is the normalized gray level of binary threshold (8 bit for HRCT and 16 bit for CT). Subscripts: *E* and *S* are for epoxy and silicone based samples respectively. *CT* is for the regular tomography acquisition (1 *pixel* = 81 *μm*), *HRCT* is for tomography acquisition at higher resolution (1 *pixel* = 1 *μm*)].

Fractality of samples was investigated by box counting technique^32^ applied on representative images of CT and HRCT slices. This method required binarized images, so the image thresholding was necessary on the gray level signals of CT slices. The fractal analyses were repeated for binary thresholding at different gray levels *i* as plotted in Fig. 4a in order to ensure unbiased results regarding the choice of thresholding. The selected *i* values matched the *i* levels determined by the automated thresholding techniques for each specimen, indicating a correct representation of the specimen morphology. Techniques are explained in the Methods section for tomography volume slicing, image treatments and fractal analyses.

#### Evidence of mass fractals

**Figure 5:**
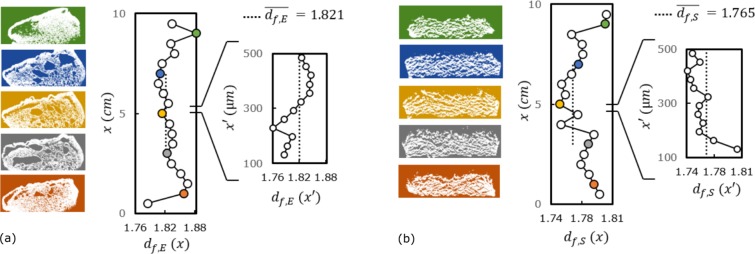
Fluctuation of fractality over *yz*-slices of: (**a**) epoxy char and (**b**) silicone char volumes. Slices are given as a function of *x* and *x*′ for CT and HRCT respectively. Power law was computed in the same range of [*a*_*min*_, *a*_*max*_] for each data point, as the example of Fig. 4b given for the middle slices. Mean fractal dimension was determined in the range of 3 *cm* ≤ *x* ≤ 7 *cm* of CT for both specimens.

Box counting technique consisted in discretizing the binarized image into voxels of dimension *a*; the numbers of voxels containing material were counted as *N*. Logarithmic plot of *N* versus *a* was plotted to observe any linear transition region indicating a possible fractality, and if applicable, to assign a fractal dimension computed from the linear regression in this region. The fractality of epoxy and silicone based samples were detected from the existence of linear transition regions on the box counting curves in Fig. 4b. Results on CT slices indicate the existence of a mass density (i.e. area density in our analyses because of the 2D problem implied by furnace testing) autocorrelation for both char samples at macro and mesoscales: both char morphologies were found to be fractal (self-similarity given in Supplementary Data). The scale-invariance was verified by HRCT (Fig. 4b). Analyses were repeated on CT slices over the range of 0 *cm* ≤ *x* ≤ 10 *cm* and on HRCT slices over the range of 100 *μm* ≤ *x*′ ≤ 500 *μm* on 3D volumes. Results are plotted in Fig. 5: slight variation of *d*_*f*_ exists over the middle volume of specimen and becomes pronounced towards the sides of specimens due to the boundary effects. The fractal dimension of the epoxy sample was found to be *d*_*f*,*E*_ = 1.82 ± 0.03, computed from the linear regression of the *N*_*E*_ versus *a*_*CT*_ curve of Fig. 4b, between the points *a* = 1 *pixel* to 128 *pixels*, for slices in the range of 3 *cm* ≤ *x* ≤ 7 *cm*. With the same approach, the fractal dimension of silicone char sample was computed to be *d*_*f*,*S*_ = 1.76 ± 0.05 from the linear regression of *N*_*S*_ versus *a*_*CT*_ curve between *a* = 1 *pixel* to 256 *pixels* in Fig. 4b for slices in the range of 3 *cm* ≤ *x* ≤ 7 *cm*. Regressions were performed over 60 data points on each specimen data set for a correct definition of their power law^33^. The error term is due to fluctuation of local fractal dimension through different discretizations *a* = 2^*n*^ at each slice.

The plotted results in Fig. 4b comply with the experimental observations. The uniform distribution of void spaces of the silicone char (Fig. 3b,g) was indicated by the uniformity of the slope of the *N*_*S*_ versus *a*_*CT*_ curve of Fig. 4b, except at the very macroscopic level where we are limited by the real sample size. As shown in Fig. 4b, linear zone of the silicone based char is larger than the linear zone of epoxy based char. For epoxy, the slope values (i.e. local *d*_*f*,*E*_(*a*_*CT*_) values) diverge gradually, indicating slightly a multifractal behavior. Indeed, the bubble size distribution of the epoxy based char is highly diverse and non-uniform from top to bottom (Fig. 3b,g) due to the different material properties and constraints at different char layers, involving a combination of bubbling, viscous movement, condensation, pyrolysis all together in the epoxy based system. The numerical analyses and the fractal description of the morphology were coherent with the visual observations.

#### Limitations and dimension scales

While theoretical fractal objects are “scale-invariant”, the fractality of real objects are only valid within a certain size range, named as cut-off. Here, size of the “fractal blocks” for epoxy and silicone formulations (*a*_*b*,*E*_ and *a*_*b*,*S*_ respectively) were defined as the upper limit of fractality: they are considered as the geometrical building blocks of macroscale samples (referring to the macro-meso-micro dimension scales in Figs 2 and 3). The upper fractal limit is expected to be either the sample size at macroscale or the maximum size of a geometrical building block at mesoscale, as depicted in Figs 2c and 3c. For a robust identification, *a*_*b*_ was determined from the upper limit of linear transition regions of box counting results of Fig. 4b on CT slices. Note that in regular CT acquisitions, each voxel (down to 81 *μm*) contained more or less a mixture of micro constituents, therefore it was not sensitive to the effect of dimension scales and image treatments. The computed values were ${a}_{b,E}^{2}\approx {128}^{2}\,pixel{s}^{2}\approx 1\,c{m}^{2}$ and ${a}_{b,S}^{2}\approx {256}^{2}\,pixel{s}^{2}\approx 2\,c{m}^{2}$, for epoxy and silicone chars respectively.

On the other hand, the lower fractal limit is generally implied by the physical and chemical constituents of the objects. For our samples, those constituents were investigated by tomography in microscale as depicted in Figs 2e and 3e. At microscale, HRCT acquisitions had a resolution of 1 *μm*/pixel, which is below the size of the constituents presented: we were able to visualize the additives and fibers on 3D reconstructions in Figs 2k and 3k owing to that resolution. The fractal results on HRCT slices (Fig. 4b) indicate around few tens of microns for the lower fractal limit *a*_*l*_ of both samples.

For silicone char (Fig. 3f,k) the isolated silicone matrix seems to exhibit slightly different fractality in the analysis of CT/HRCT: at high threshold levels *i* (Fig. 4a), the light elements as carbon disappear on CT/HRCT images but a certain fractal dimension *d*_*f*_ is kept constant for the remaining signals representing the silicone binder blended heavier components. This indicates the existence of an organized sub-network due to the silicone based polymeric matrix. This sub-network exhibits a secondary fractal dimension *d*_*f*,*S*,*secondary*_ ≈ 1.65, which indicates the time-dependent (re)organization of silicone based binder in (2D) space, apart from the overall network. This dual fractality proves the two-step mode of action regarding the time-dependent expansion of the silicone based coating. The dimension *d*_*f*,*S*,*secondary*_ is attributed above microscale; therefore it is related to, but doesn’t give the exact value of, the intrinsic behavior of the molecular polymeric network^34^. For this reason, the following sections will reflect the effective medium (conceptualization/simulation) for the constituents of both coating types, rather than any molecular organization.

For silicone based sample, if one needs to consider the effective medium properties of the whole coating formulation at mesoscale (i.e. silicone matrix blended with EG), the smallest geometrical cells are limited by the maximum size of the expandable graphite platelets. The commercial EG grade used in our study can include platelets up to a size of 800 *μm*^35^ and the expanded worms on Fig. 3f,k,o validated this information. This value falls within the region around *a*_*u*,*S*_ ≈ 8 to 12 *pixels* ≈ 650 to 970 *μm* in the fractal region of silicone char indicated with an arrow on the *N*_*S*_-*a*_*CT*_ curve of Fig. 4b. With a similar approach, unit cells of the fractal block of epoxy sample were determined according to the longest length of fiber-like constituents from hundreds of microns up to a millimeter as depicted in Fig. 3e,k. This value falls within the region around *a*_*u*,*E*_ ≈ 8 to 12 *pixels* ≈ 650 to 970 *μm* in the fractal region of epoxy char indicated with an arrow on the *N*_*E*_-*a*_*CT*_ curve of Fig. 4b. Therefore the unit cells of the fractal blocks were defined as *a*_*u*,*E*_ ≈ 900 *μm* and *a*_*u*,*S*_ ≈ 800 *μm*, for epoxy and silicone chars respectively. Note that for macroscale applications, the fractality between [*a*_*u*_, *a*_*b*_] will ensure the “fractal density” whereas the fractality between [*a*_*l*_, *a*_*u*_] will determine the “fractal tortuosity”. In the next sections, the implications of the above numerical results will be presented for a better understanding of fire testing results. Possible ways of virtual conceptualization will be discussed: an intersection between our fractal patterns and random movement generators will be researched to mimic the intumescent modes of actions by virtual building blocks, in order to simplify the problem of coating expansion.

### Implications and conceptualization of expansion

**Figure 6:**
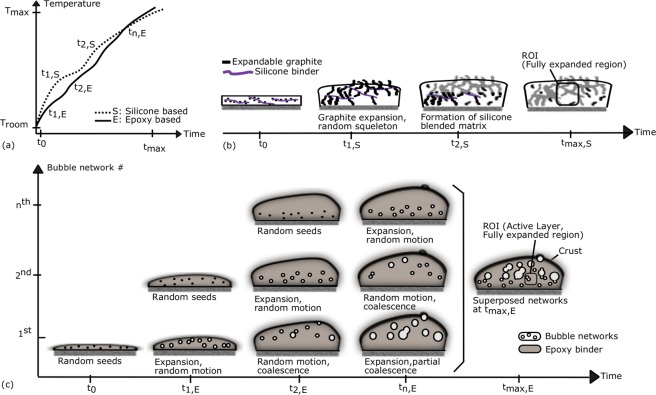
Schematic illustrations of intumescent coating performances and expansions. (**a**) Dimensionless plot of the performance curves; temperature is mesured on the backside of steel plates as a function of test time. Time *t*_0_ accounts for the virgin coating in the beginning of the fire testing; *T*_*room*_ is the ambient temperature; *T*_*max*_ is the maximum test temperature against the structural failure of steel, generally chosen between 400 °C and 500 °C; subscripts *S* and *E* account for silicone and epoxy respectively. (**b**) Our resulting concept of the expansion steps for silicone binder formulations with expandable graphite. (**c**) Our resulting concept of the expansion steps for epoxy binder formulations with bubbling actions. ROIs are the regions of interest, selected in the active char layers.

In this study, the expanded pattern of two distinct intumescent systems exhibit very similar fractal dimension of around 1.8, despite their difference in chemical formulation and in their heat induced reactions. Many physical objects and phenomena exhibiting fractal behavior were shown to be virtually reproduced by numerical algorithms based on autocorrelation functions and/or random generators^36^. Consequently, is it possible to use the same numerical algorithm to generate artificially the virtual morphologies of samples (question never tackled in literature on intumescence) despite the visual evidence of dissemblance? Here, two intumescent systems have different “expansion actions”. Therefore, the answer lies in the intumescent modes of action of coating formulations, because the mathematical models can interpret differently the random mechanisms attributed either to the “medium” or to “movement” depending on the physical context^37^. Preliminary applications will be presented in the next section using random algorithms. Beforehand, it is necessary to understand the mechanisms of formation, even though in the final stage (i.e. in the numerical generation of virtual geometry) the kinetics will be omitted. Thereby, we will investigate the randomness of the coating expansion. To that end, the temperature-time curves issued from fire testing results were conceptualized together with the reported experimental data on similar modes of action with epoxy^7^ and silicone^29^ binders respectively and illustrated accordingly in Fig. 6a. The curves represent the typical patterns of temperature increase of coated steel plate versus heat exposure time during fire testing. Plots were presented after normalizing them by the maximum test temperature and time, because we aim at establishing a generalized conceptual solution, and if applicable, with dimensionless parameters. Based on the reported data, the fractal analyses bring new evidence and have implications for the understanding of the characteristic expansion steps, here indicated by the time steps *t* (*t*_*S*_ for silicone char of Fig. 6b and *t*_*E*_ for epoxy char of Fig. 6c).

#### Random bubbling

Earlier researches modeled the classical intumescent systems as the growth and movement of bubbles in a hot oversaturated liquid-gas solution^19^, limited to 1D solutions for morphology, due to complexity of multiphysics simulations. A simplified assumption was the summation of the fields around individual bubbles^10^. Inception of seeds occur when the temperature inside the specimen increases enough and when there are still reactants to form blowing agents causing bubble formation. New bubbles are created by further propagation of heat toward the steel plate. Accordingly, the distinct time steps from *t*_1,*E*_ to *t*_*n*,*E*_ (Fig. 6a and literature data therein) shall be related to the retardation of heat propagation inside the expanding specimen due the inception of new bubble networks together with the expansion of the existing ones^10^. Hence, the epoxy coating expansion was conceptualized as a superposition of randomly distributed incipient bubble networks formed at successive time steps as illustrated in Fig. 6c. This claim is partly supported by the fractal analysis in Fig. 4c which indicated slightly multifractal behavior of epoxy char at variable size scales. On the other hand, bubbling of thermoplastics in fire was modeled as a diffusive motion, followed by the coalescence and expansion, resulting in the explosion of bubbles close enough to the free surface^38^. For intumescent coatings, the bubbles close to the surface cannot always explode due to charring and condensation of the top layer. This induces mechanical squeezing due to the solidified crust (Fig. 6c). Hence the conceptualized ROI (fractal building block) is to be selected in the active layer behaving as a “viscous medium” at the bottom: bubbles were considered in diffusion limited motion in this surrounding fluid. This claim is supported both by CT data and fractal results. The pores with low aspect ratio (considered as fresh bubbles) were mostly situated in the bottom part of the char (illustrated in the Supplementary Video V6). Size of those pores fall in the region indicated by unit cell arrow of *N*_*E*,*CT*_ versus *a* curve in Fig. 4c. The fractal results comply with conceptual model (even though the fractal analyses were performed on the solid pattern and not on the pores, because the viscous char followed naturally the growth of bubbles). Therefore, on Fig. 6c, the ROI (region of interest) for conceptualization was sketched in the active layer of a fully expanded region.

#### Random skeleton

The results of fire testing and the reported data indicate two major characteristic time steps for silicone binder coatings as sketched in Fig. 6a. It was explained in the experimental results that the low temperature first stage *t*_1,*S*_ corresponds to the creation of “worms” due to sublimation of blowing agents at lower temperatures (around 200 °C). This leads to the formation of a random expanded EG skeleton, probably dragging most of the silicone binder away from steel plate, as sketched in Fig. 6b. Polymeric matrix penetrates in the empty sites around EG worms. This is not only physical but is also due to chemical bonding, forming a regular structure as fractal analyses indicated a second fractal nature of the silicone binder (Fig. 4a). This shall be the result of the reorganization of the degraded polymer matrix due to crosslinking^39^ following the EG squeleton and probably reacting with EG flakes^29^ (Fig. 3o,p), leading to a gelation-like polymer matrix^40^ as an organized 2D/3D structure (in our case in 2D slices) formed when the expansion coefficient reached its limit leading to charring of the fire protective coating^5^. Consequently, the second stage *t*_2,*S*_ is attributed to the fractal behavior where silicone matrix and/or its decomposition products were presumed to penetrate the pores. Unlike epoxy formulation where the bubbles were in motion, here the movement was restricted by the char material itself (EG). Therefore the randomness is attributed to the medium, i.e. to the random skeleton formed by the formation of graphite worms where the silicone binder is obliged to penetrate and bond to the non-occupied sites. The resulting geometrical building blocks are large and more or less uniform, containing the graphite flakes and worm-like structures. The fractal analyses, together with tomography data, constitute a visual and conceptual proof of the self-expansion and percolation of viscous matrix which was partly presumed in former studies.

### Immediate application: generations of virtual morphologies

#### Building block of epoxy based system

**Figure 7:**
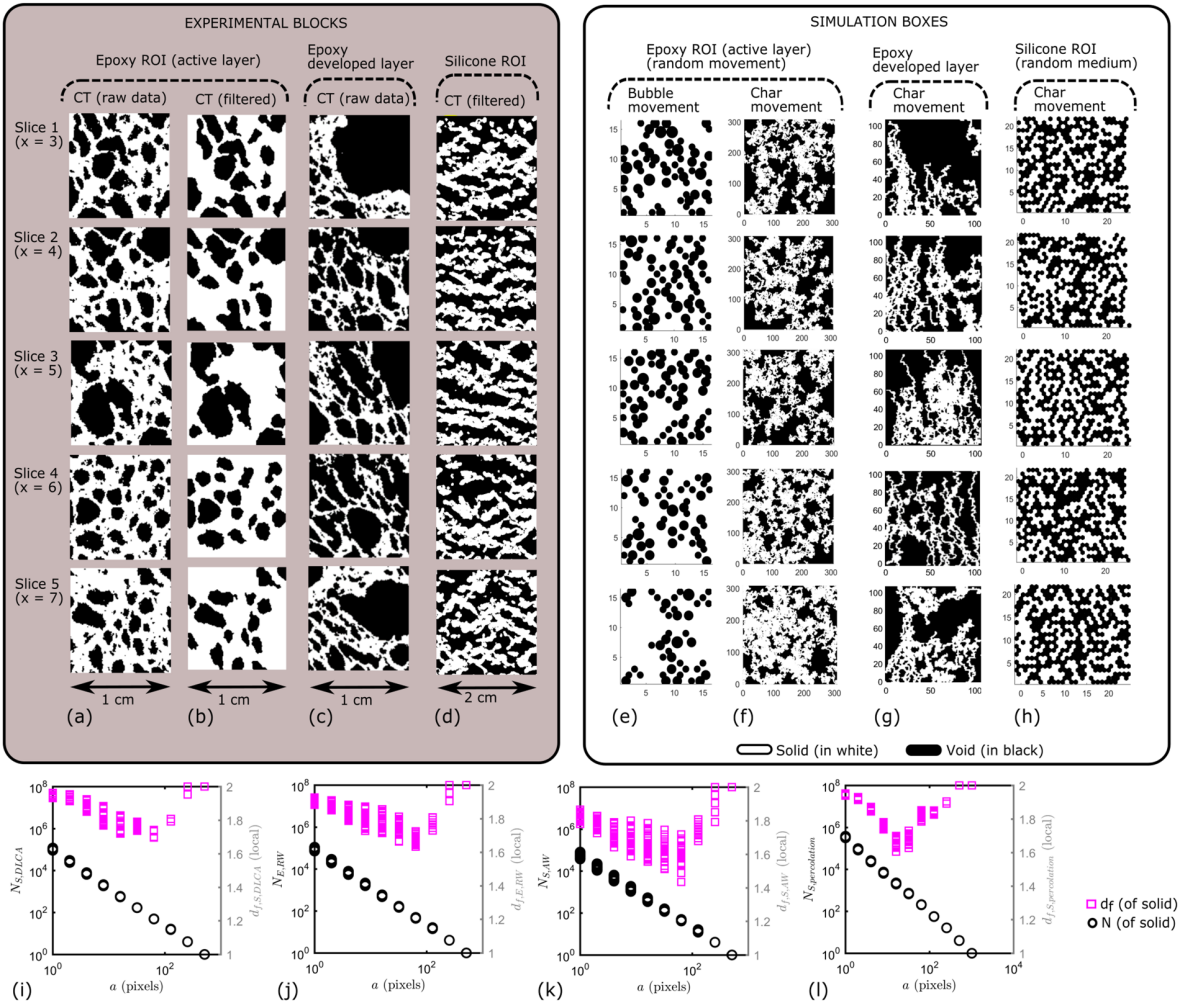
Building blocks of intumescent specimens. (**a**–**d**) CT slices. ROI was selected in the active char layers as they are the most representative parts of the modes of actions of the coatings. For epoxy coating, developed char layer was also simulated referring to different material properties. (**e**–**h**) The equivalent virtual geometries were simulated by DLCA-like, random walk, active walk and site percolation algorithms respectively, based on data of the building blocks analyzed in Fig. 4b. The size of blocks was determined as indicated by the size of the fractal blocks *a*_*b*_. The size of the elementary blocks (distance between the lattice nodes) is equal to *a*_*u*_. The CT image data is used for the computation of void ratio *e*, in order to complete the morphology parametrization (i.e. *a*_*u*_, *a*_*b*_, *a*_*l*_, *d*_*f*_). (**i**–**l**) Post-processing of simulation boxes, virtual mean *d*_*f*_ values computed from linear regressions between 2^nd^ and 8^th^ data points.

The epoxy char is a bubbling system and the nucleated bubbles were presumed to follow diffusive motion in viscous char (Fig. 6c). As the fractal results indicate autocorrelation of geometry, randomness needs to be assigned to a specific repeated pattern for conceptualization. Here, the “randomness” shall lie in the “inception pattern” and the movement can be assigned either to the bubbles or to the walls. Following the fractality given by box counting results, the random repartition of bubble initiation and growth in intumescence driven by diffusion^10^ can reasonably be simulated by a random generator based on a diffusion limited model, replacing a bubble network as particles in motion surrounded by a viscous fluid^37^. Each bubble network (Fig. 6c) was conceived as a fractal aggregation pattern simulated by a DLCA-like (diffusion limited cluster aggregation) algorithm followed by (partial or total) coalescence of the bubbles. Note that the fractal aggregation and random walk algorithms are capable of providing accurately enough the “statistical distribution in space” of a material exhibiting fractal similitude properties^41^. Consequently, the geometrical building blocks (Fig. 7a,b) were reasonably simulated for the active char layer as presented in Fig. 7e. Number of occupied sites was estimated from the void fraction Φ of filtered CT data of Fig. 7b. Each CT slice exhibit slightly different Φ and *d*_*f*_; 6 random generations were performed per slice, resulting in a total of 30 independent random generations. Those 30 virtual geometries exhibited a mean fractal dimension of *d*_*f*,*E*,*DLCA*_ = 1.78 ± 0.07 as computed from the plot of Fig. 7i.

Alternatively, the random motion can be assigned to the pore walls. In this case, we need to perform multiscale simulation: the main mesh contained the random seeds (random bubbling) and the motion of the pore walls were simulated by random walk (RW) in a submesh leading to a tortuosity as presented in Fig. 7f. The number of seed and the walk steps were estimated from Φ of raw CT data of Fig. 7a. Virtual geometries exhibit a mean fractal dimension of *d*_*f*,*E*,*RW*_ = 1.77 ± 0.07 as computed from the plot of Fig. 7j. Slice by slice simulations give void fractions and *d*_*f*_ values close to the ones of the real object, which supports our conceptualization approach on the action of epoxy based coating.

Although the main scope of study is to mimic the active intumescence, we briefly identify regions located above active layer. Developed layer (between ROI and crust in Fig. 6c) exhibit different material density and structure on CT slices (Fig. 7c) and its motion shall not be represented by RW. Reasons are twofold: material properties differ in terms of phase/composition and viscosity with evolving temperature and time; condensation and charring leads to the formation of a crust. Under the crust, layers are squeezed, some walls are broken or pyrolyzed with entrapped gases. While the active layer leads to continuous expansion, movement of developed layer is somewhat restrained by the evolution of intumescence over time with expansion ratio, charring rate, formation of crust. Such complex phenomena can be best described by an active walk (AW) model^42^. (Note: *active layer* (ROI) of char is not to be confused with *active walk*.) The immediate use of AW is inconvenient for the simulation of whole char morphology because AW makes use of potential fields. In our case, this involves transient and non-homogeneous material properties as function of internal heat propagation (and eventually pressure fields). At present, non-intrusive *in*-*situ* monitoring of those fields is impossible, so, we don’t have realistic data. We made hypotheses for a preliminary application of AW to a block of developed layer, in the fully expanded region, in order to show that our fractal virtual concept shall fit distinct intumescent actions. Yet, the algorithm shall converge to RW for the ROI of char shown before (see Methods). A mean dimension of *d*_*f*,*E*,*AW*_ = 1.73 ± 0.10 was exhibited by the resulting building blocks of AW (Fig. 7g,k) over 5 slices, due to higher ramification and lower material density in this layer of char.

#### Building block of silicone based system

In the conceptualization stage, the final fractal morphology was attributed to the penetration of the silicone binder in a random skeleton formed of expanded graphite. The randomness needs to be assigned to the “medium” in which the viscous char is moving. This mechanism shall be best described by percolation^37^. Here we propose the simulation by site percolation on an triangular lattice with a probability of having open/close sites of a lattice: in physical terms, this lattice is mainly formed by the expanded EG skeleton due to the first expansion step *t*_1,*S*_ (Fig. 6b). The site occupation probability was estimated from tomography slices as the void fraction (i.e. the number of pixels containing materials to the total number of pixels). The CT computed probability of finding material was *p* = 0.50 ± 0.03. The error term is estimated from images with and without denoising. The result is quite interesting, as *p* = 0.5 is the critical value of site percolation on triangular lattice^43^ in order to form an infinite connected structure, noting that the critical percolation clusters exhibit fractal structures^43,44^. Each CT slice exhibit slightly different *p*: 6 random generations were performed for each CT slice resulting in a total of 30 independent generations to represent the same specimen. One example for each slice is presented in Fig. 7h and compared to the CT equivalent of Fig. 7d. The virtual geometries were post-processed by fractal analyses and they exhibit a fractal dimension of *d*_*f*,*S*,*Virtual*_ = 1.75 ± 0.07 as computed from the plot of Fig. 7l. This value is very close to the fractal dimension of the real object which was computed as *d*_*f*,*S*,*Real*_ = 1.76 ± 0.05, which supports our conceptualization of the action of silicone based/EG system.

#### Prospective insights on fire modeling

Some universal validity of our approach was confirmed toward establishing a generalized solution of expanded intumescent geometry by use of fractals. It consituted a complementary alternative to the existing pore definition approaches^11^, brought new evidence to intumescent mechanisms, and can be extended in the future to define virtual 3D porosity^11^ owing to the validity of 2D problem presented in this study. Thanks to the scale-invariance of the char fractal property down to *a*_*l*_ ≈ 30 *μm*, the above virtual generations can be further exploited in order to include microporosity (smaller than *a*_*u*_) with their wall tortuosity by incorporating submesh fractals into the mesoscale building blocks. This was partly demonstrated by the RW and AW generations for the epoxy based system (Fig. 7f,g). In particular, AW seem to be a powerful tool for future studies in order to explain, for example, the thermal-mechanical resistance of intumescent specimens under variable fire constraints by playing with the self-evolution of landscape (in Supplementary Information).

The proposed concept can serve as a predictive tool for the physical effective properties and for the evolution of morphology through coating expansion, as it was initially required for a correct predictive intumescent modeling contrary of using only the expansion ratio as an input^45^. It can possibly be incorporated in global modeling tools through semi-empirical relationships because it can reasonably be assumed that fractal morphology of the active layer will be preserved regardless of the expansion ratio and throughout the coating expansion due to random nature of events: the physical properties will only be temperature dependent in the active char layer, hence reducing partially the uncertainty in the time-dependent effective properties of the coating throughout its fire reaction and expansion. Alternatively, the numerical models of intumescence could be divided into sublayers, each having a thickness of *a*_*b*_ represented by a fractal building block: the active layer would be a static “fractal” layer, the number of charring top layers could be predicted from the initial thickness of the virgin coating. Those proposed concepts shall be studied in prospective analyses.

## Conclusion

In this study, a new analysis technique is applied to the condensed phase residues of fire protective intumescent coatings: expansion evolution and expanded morphologies were conceptualized by use of fractals. To that end, the inner morphologies of charred residues were computerized after fire testing. Two different systems were analyzed, i.e. bubbling and self-expanding intumescence representing chemical and physical modes of actions respectively. Mass fractal behavior was observed for both expansion patterns. This brought new evidence to the chemical and physical actions of constituents, with possible reflections on the heat barrier properties of coatings during fire testing. We demonstrated a first fractal parametrization and virtualization of the fire protective intumescence between scales of few centimeters down to tens of microns. Such a mathematical parametrization was a powerful tool, because random algorithms were capable of numerically generating virtual building blocks of expanded coatings, proving some universality of the technique to intumescence. This latter finding is promising as it can be used further in coupled modeling tools and simulations for fire. Furthermore, a first demonstration was also presented by use of a more generalized model (AW), toward establishing the overall intumescent morphology and charring, which can lead in the future to a realistic simulation of intumescence without use of physical or chemical models. Up to now, to our knowledge, none of the multiphysics simulations was capable of simulating the intumescent expansion in such realistic patterns.

Ideally, an *in*-*situ* X-ray tomography would be necessary in order to establish quantitative correlations between the time-dependent properties. This was inconceivable due to the engineered standard of fire testing. It shall be studied in future works, either using a new design of fire testing ensuring similar external constraints, or using different physics to observe the inner morphology. Then, the physical properties, such as the mechanical or thermal resistance, can be correlated to fractal parameters by semi-empirical relationships; direct modeling of the latter can reduce the uncertainties and errors in effective medium properties and in inverse problems for heat transfer modeling and fire simulations. To go beyond the upper fractal limit observed, coupled simulations can be used to describe macroscale behavior such as the mechanical squeezing effects and heat induced pyrolysis at different scenarios leading to char degradation beyond 400–450 °C. Similarly, lower fractal limit can be extended below the micron scale by tackling the physical and chemical interactions between elementary constituents down to molecular structures. On the other hand, fractal clusters have been and are still subject to many researches, and of interest for the variable definition of more robust mathematical models. Therefore we presented a step forward linking some fundamental aspects to applied research on materials science in fire safety engineering, with practical results and prospective insights.

## Materials and Methods

### Intumescent specimens

Charred specimens were obtained from fire-protective intumescent coatings exposed to bench-scale hydrocarbon fire testing. Two types of coatings were selected to represent different intumescent actions: an epoxy based formulation (containing bubbling agents for expansion) and a silicone based formulation (with expandable graphite for expansion). Samples for coatings, resins, catalysts, and primers were supplied by Dow Corning (DC, Belgium) and Advanced Insulations Systems (AIS, United Kingdom). All samples were supplied as two-part system of a base and a catalyst/curing agent. The epoxy-based system was a commercial formulation^29^ in two part system and the silicone-based system was a partly in-house mixture^29^ containing silicone binder, expandable graphite and glass fibers. Steel plate preparations, coating and curing were accomplished according to reported experimental protocols both for epoxy and silicone based mixtures respectively^7,29^. The only difference was the expandable graphite rate in the silicone mixture, using EG with 80–98% carbon (Asbury). The coating formulations were applied on steel plates of 100 mm × 100 mm × 33 mm, cleaned priorly with a scrubber, washed with water and degreased with ethanol. Dried plates were cleaned by sand blasting. Anticorrosion pre-treatments were applied by plunging the plates into a phosphatation bath prepared according to the reported procedure^46^. Interzinc or Epodux 57–35 primer was coated on steel plates before applying epoxy-based coating and OS1200 primer was coated before applying the silicone-based coating^7^. Base and curing agents were in the ratio of 10:1 and were mixed at room temperature just before applying the coating. After mixing, steel plates were coated with the prepared fire protective intumescent coatings on one side. The coating thickness was 6 mm for epoxy formulation and 4 mm for silicone formulation. Coated plates were cured for 24 h at room temperature followed by another 24 h at 40 °C.

### Fire testing

The cured specimens were subjected to furnace test mimicking UL1709 normalized temperature versus time curve, simulating hydrocarbon fire at bench scale^7^, following the reported protocols^39,47^. The furnace exhibited an internal volume of 26 *dm*^3^ and the refractory fiber panels, stable up to 1300 °C, covered the internal walls of the furnace as was sketched in Fig. 1a. The furnace was equipped with two propane burners of 20 kW. The gas pressure was fixed at 1.8 bar and the flow was regulated in order to mimic the UL1709 curve. A temperature probe inside the furnace regulated the temperature and a thermocouple allowed the furnace temperature profile to be registered. The temperature was measured at the backside of the specimen plate using a pyrometer (temperature measured in the center of the plate). The backside of this steel plate was coated with black paint having a constant emissivity of 0.92 and thermally resistant up to 800 °C. The fire testing was stopped when the measured backside temperature of the plate reached 500 °C, the adopted standard value for normally loaded steel structural components^48^. Once the samples were cooled down, the expanded and charred specimens, i.e. the intumescent chars, were collected together with their steel plate.

### Tomography and imaging

The expanded intumescent specimens (i.e. the intumescent chars in Figs 2a and 3a) were placed in X-ray *μ*CT (computed microtomograpy) for the analysis of the inner morphology without structural damage. The resulting recordings over 360° were reconstructed to obtain the computerized 3D CT volumes (Figs 2i and 3i). After regular CT acquisitions, char samples were cut thoroughly (Figs 2b and 3b) for visual inspection through their mid-sections with a cutter, and the cross sections were compared at multiscale. The inner structures were analyzed by correlative observations between computed and optical means. Tiny specimens were collected in the middle of chars (Supplementary Fig. S1) to obtain 3D HRCT (high resolution CT) volumes (Figs 2k and 3k). The tomography was performed using the microtomography setup at ISIS4D X-ray CT platform^49^ (equipped with UltraTom from RX Solutions). The set-up consisted of two X-ray tubes (micro and nano focus), a sliding and rotating stage holder, a flat panel detector (1920 * 1496 px − 127 *μ*m/px − 0.2 to 60 frame/s), a linear detector (2560 px − 200 *μ*m/px − 0.2 to 60 frame/s), a CCD camera (4000 * 2624 px − 11.8 *μ*m/px-up to 3.4 frame/s) and an image intensifier. Samples were placed on styrofoam holders and then mounted on the rotating stage to minimize the signal noise due to the holder. Samples were rotated by 360° with an angular step of 0.25°. The subsequent tomography volumes were reconstructed from 1440 projection images produced by classical attenuation contrast technique and visualized using X-Act (from RX Solutions). Reconstructions had a resolution of 81 *μ*m/voxel (CT) and 1 *μ*m/voxel (HRCT). Before the CT, the silicone-based sample was detached without damage from its steel plate and placed on styrofoam directly. The epoxy char had to be placed with steel plate due its high adhesion to surface. Hence, a tiny part of the information was very noisy on CT of the epoxy sample due to scattering of some X-rays from steel plate on the bottom of the sample. Thereby, analyses were performed by omitting this small noisy bottom region from tomogram reconstruction. As this section was relatively small compared to the whole volume, its exclusion would not change the overall fractal dimension, if any, upon numerical analyses, with the condition of certain scale-invariance and self-similarity: it would slightly shift upwards the plot *N*_*E*_ vs. *a* (Fig. 4b) without changing the slope (i.e. *d*_*f*_) due to a slight increase of the total number of voxels. The specimens were very crumbly, hence small samples needed to be embedded into an epoxy resin to keep the structural integrity of the sample in electron microscopy chamber for chemical mapping. Embedded samples were cut, polished (down to 1/4 *μm*) and subsequently imaged by optical-digital microscope. Then, samples were carbon coated with a Bal-Tec SCD005 sputter coater. A Cameca SX100 electron probe microanalyser (EPMA) was used to perform elemental analysis. Back scattered electron (BSE) images of the cross sections were obtained at 15 *kV* and 15 *nA*. Chemical profiles were mapped at 15 *kV*, 40 *μA*.

### Image treatments

The cuts of the real specimens were compared to the ones of the computerized tomography slices. Image treatments were performed on the computerized volumes of chars. CT slicing was performed according to the 2D problem implied by the furnace testing (Fig. 1a). In fire tests, the flame was oriented along the vertical axis in Fig. 1a, which corresponds to the y-axis of tomography reconstructions. Hence, the boundary conditions are supposed to be identical for slices parallel to yz-planes. The computations were repeated for vertical slices of the char to check any anisotropy of the problem in x-direction. Slices with *x* = 3, 4, 5, 6 and 7 cm were considered in order to minimize any cooling boundary effects that may occur in furnace test conditions. The pore interconnectivity for semi-correlative observations (Figs 2g and 3g) was visualized in ImageJ by using gray level watershed algorithm without any filtering/blurring^50^. Our tomography acquisions were performed by absorption contrast. One difficulty is to ensure the maximum visibility of carbon elements in our charred samples because low atomic number elements interact only little with X-rays and tend to produce low gray levels susceptible to be confused with reconstruction noise in the image signal histograms^17^. The technique can lead to uncertainties and errors up to a factor of two for microscale tortuosity according to uncertainty levels arising from binarization threshold and image filtering^51^. In our study, the only filtering was performed right after 3D reconstruction: variance filter was applied as it is the minimum filter required to remove noise on 3x3x3 voxel volume^51^. After binarization, all the computations were performed through fractal dimension. i.e. through the density distribution of surface area on slices (cross-sections of expanded coatings) and not through the tortuosity. Therefore, the uncertainties were reasonably minimized only to thresholding. For the fractal area estimation, the “intermean” and “moments” methods were used to treat the signal histograms of stacks. For regular CT (1 pixel = 81 *μm*), the automated IsoData of ImageJ^52^ (“iterative intermeans”) was used because it was reported as being one of the robust techniques for microtomography image treatments of engineered materials^53^. It worked relatively well on CT volumes because the samples were rather homogeneous and gave distinct dual peaks (“background peak” and “material peak” on the plot “pixel number versus gray value”). Those peaks were not clear enough for ISO50 on the HRCT (1 pixel = 1 *μm*), probably due to noises arising from: the ring artifact (high magnification with erroneous pixels), a slight rotation axis error (due to precision limits of the rotating equipment supporting a very tiny tomography sample), and some scatter artifacts (due to heavy elements of additives in the coating samples). The variability in the elementary composition of specimens was less important for the standard CT where each pixel contained a combination of materials. For HRCT, it became more problematic as each pixel was capable of representing rather uniform chemical composition: carbon was blended with elements having much higher atomic numbers. As a result, ISO50 thresholding method was not capable of detecting correctly the material edge for HRCT. For the latter, the global “moment-preserving thresholding”^54^ had shown the best performance on noisy/fuzzy images^55^: this resulted in less noisy BW versions of HRCT stacks, and preserved the mass density of materials for their fractality (if any) through subsequent numerical analyses as the algorithm preserves the gray-level moments while thresholding.

### Numerical analyses

The analyses of the char tomography were performed by means of fractals. Box counting^32^ was applied on binarized slice stacks of CT and HRCT volumes. This technique consisted of covering the slice image with voxels (boxes in 2D). The fractal dimension *d*_*f*_ was computed following the correlation given:

1 $${d}_{f}=\frac{log(N)}{log\mathrm{(1}/a)}$$

where *a* is variable box dimension in pixels (which evolves practically as a power of 2) and *N* is the minimum number of boxes needed to encompass the whole object that contained the material. The box covering was performed on the surface area and not only on the surface boundaries as we are interested in the density autocorrelation not limited to tortuosity. The curve *log*(*N*) versus *log*(*a*) was plotted; if any linear transition region was observed then the fractal dimension was equal to the slope of the curve. Note that the maximum value of fractal dimension with this method can be equal to a maximum slope of 2 in 2D (or slightly lower in practice as the numerical methods saturate^32^). The algorithm was applied following reported procedure^56^ previously benchmarked on virtual fractal volumes simulated by DLCA (Diffusion Limited Cluster Aggregation) simulations and on experimental fractal volumes obtained by electron tomography^57^. Algorithms were written in C++ and Matlab^58^ to analyze image stacks from *μ*CT slices, covering large spectra of signal thresholding. Finally, fractal analyses were repeated at different thresholding levels of tomography signals for two reasons: first, in order to ensure that the results are invariant through slight changes in image intensity, and second, in order to check the fractality of different chemical components yielding information about the intumescent kinetics (Fig. 4a). Analyses were performed from macro (around 10 cm of sample size) down to micro scales (1 pixel = 1 micron on HRCT slices) to check the scale invariance. Self-similarity was checked on CT slices (provided as Supplementary Data).

### Numerical simulations

Following the results of the fractal analyses, geometrical building blocks of porous-like chars were generated in 2D space using algorithms based on random generators. Note that a specific phenomenon or morphology can only be described by a certain range of fractal dimensions where the physics are implicitly defined and still, the same fractal dimension can be attributed to many different systems or objects^59^. So, the randomness was attributed relevant to the observed and the reported experimental data in terms of the intumescent modes of actions as summarized in Table 1. For numerical generations, the input data was partly determined by fractal analysis results and partly fed by CT data.

**Table 1:** Overview of methodology for numerical generation of virtual geometries.

Sample type:	Epoxy based	Epoxy based	Epoxy based	Silicone/EG based
Random attribution:	Movement (foaming/charring)	Movement (foaming/charring)	Movement (foaming/bubbling)	Medium (self-expansion/physical percolation)
Moving object:	Walls (viscous char)	Walls (viscous char)	Pores (bubbles)	Walls (viscous char)
Simulated by:	Random walk	Active walk	Aggregation-like	Site percolation
Inception scale:	Meso - mesh (seeds - square lattice nodes)	Meso - mesh (seeds - square lattice nodes)	Meso - mesh (seeds - square lattice nodes)	Meso - mesh (EG sites - triangular lattice nodes)
Movement scale:	Micro - submesh (char - square lattice)	Micro - submesh (char - square lattice)	Meso - mesh (bubbles - square on-lattice)	Meso - mesh (char - hexagonal elements)

#### Bubbling system

**Figure 8:**
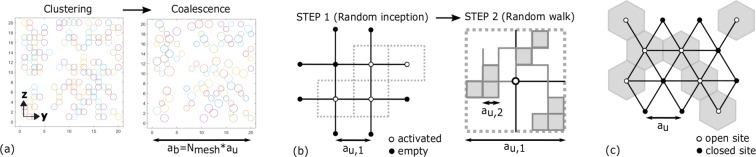
Schematic illustrations of simulations. (**a**) Random clustering (DLCA based) for epoxy char bubbles; (**b**) Random movement for viscous epoxy char; (**c**) Site percolation for silicone char.

The random distribution of bubble clusters of epoxy type char was simulated using a DLCA-like (diffusion limited cluster-cluster aggregation) algorithm because it is capable of generating clustered objects with a mean fractal dimension of around 1.7 in 2D space^36^, more extensively between 1.5 and 1.9 depending on the simulation conditions, packing density, cluster size, system size and constraints of clustering^25^. Here, groups of bubbles were conceived as clusters and the original numerical model^60^ was implemented: both the individual seeds and clusters were mobile, clustering was irreversible and random on the nodes of a square lattice with periodic boundary conditions. Random initial seed was followed by random movement until all the seeds aggregated in clusters (i.e. until the ultimate random distributions of bubbles were obtained). The total number of activated sites was given according to the void fraction (i.e. void surface area fraction in 2D), computed in ImageJ using particle analyzer masks of CT data. Simulations were performed on-lattice for simplicity, because differences are negligible on- or off-lattice for few hundreds of activated sites^25,36^. Finally, our simulations differed slightly from the classical technique^36^ because the density of initial seeds was quite high up to 35%, the fractal dimension was not imposed a priori in order to keep the simulations thoroughly random, effect of mobility was not taken into account. DLCA-like clusters were post-processed to coalesce the neighboring bubbles conforming to the pore-connectivity (observed by watershed) and pore-circularity (observed by particle counter masks, Supplementary Video 6). Bubbles on adjacent lattice sites were coalesced to form larger bubbles and the operation is performed from +y to −y (left-to-right of char slice, mechanical constraint) and from +z to −z (top-to-bottom of char slice, thermal gradient), as illustrated in Fig. 8a. In this work, we simulated the morphology of active char layer. Consequently, other physics are reasonably ignored because they are not primary modes of actions in this layer, such as the mechanical squeezing of bubbles due gravity at high expansion ratios, or partial coalescence by further pyrolysis of the pore walls at long exposure times to flame. This operation can be further iterated to other regions of the expanded coating depending on the local constraints during expansion and on the selected size of building blocks, which needs to be tackled further by coupled physics.

Alternatively, epoxy char walls were also simulated by random generation on multiscale mesh as illustrated in Fig. 8b. In step 1, coarse mesh (where ${a}_{b}={N}_{mesh}\,\ast \,{a}_{u,1}$) sites were activated randomly representing the random seeds; distance between lattice nodes is *a*_*u*,1_ = *a*_*u*,*E*_. Then in step 2, each site was divided in a submesh and the pore walls were traced by random walk (Fig. 8b); *a*_*u*,2_ is selected slightly larger than lower fractal limit *a*_*l*,*E*_. Both the number of seeds and the number of walk steps was estimated from the void fraction of CT slices. The technique is inspired from the multi-step fractal generation proposed by Jin *et al*.^59^; yet, it is slightly different because we didn’t impose a priori *d*_*f*_ to keep the process thoroughly random, and the activated sites of step 1 are not completely filled. Therewithal, the parameters our simulations are compared to their theoretical equivalent as summarized in Table 2 in which the fractal scaling proposed by Jin *et al*. is defined as:

2 $${d}_{f}=\frac{log\,F}{log\,P}$$

where *F* and *P* are the “dimensionless scale-invariant” properties of a fractal topology: *F* is the “scaling coverage” given by Eq. (3) and *P* is the “scaling lacunarity” given by Eq. (4). Equations were adapted from reported theory^59^ as follows:

3 $$P={l}_{1}/{l}_{2}$$

4 $$F=M(S{S}_{2})/M(S{S}_{1})$$

where *SS*_1_ is the “step 1” simulation and *SS*_2_ is the “step 2” simulation; *l*_1_ and *l*_2_ are the characteristic dimensions of mesh cells in *SS*_1_ and *SS*_2_ respectively. This representation with *F* and *S* was mathematically proven to be valid for a same fractal topography^59^, independent of the geometry/size/dimension of the fractal object defined. Thereby, at each step, a different generator can be used without affecting the fractal scaling result. Indeed, the physical applicability of this concept is clearly proven by our random generations of epoxy char morphology in Fig. 7e. Also, it explains clearly the high dispersion and some overestimation of *d*_*f*_ (see effective *d*_*f*_ in Table 2) in our simulation boxes (Fig. 7h) compared to the experimental value of *d*_*f*,*E*_.

**Table 2:** Two-step virtual generation of the epoxy based sample: step 1 simulation (SS_1_) is random inception and step 2 simulation (SS_2_) is random walk.

Slice number	Void ratio	*M* (*SS*_1_)	*M* (*SS*_2_)	*P*_*simulation*_ (eqn. 3)	*F*_*simulation*_ (eqn. 4)	Effective *d*_*f*_ (eqn. 2 with *F*_*simulation*_)	*F*_*theoretical*_ (eqn. 2 with *d*_*f*,*E*_ = 1.82)
1	0.476	52	4663	11	89.7	1.87	78.6
2	0.481	52	4618	11	88.8	1.87	78.6
3	0.492	51	4433	11	86.9	1.86	78.6
4	0.404	60	6119	11	102.0	1.92	78.6
5	0.327	67	7716	11	115.2	1.97	78.6

M is the number of activated cells for each steps, estimated from the void ratio of the slice.

As a third alternative, the movement of walls in the developed layer (Fig. 7c, between ROI and char crust) were simulated by a random bubble inception followed by probabilistic active walk (Fig. 7g). The walker moved to adjacent sites in a guessed (yet unknown) potential field where the sum of potentials shall be equal for the blocks in each slice, with a stepping rule presumably proportional to 1/Δ*T* (*T* is temperature) (a discussion on model parameters is given in Supplementary Information). It is noted that AW converges to RW in ROI (active layer) of char: ROI inception and growth occur in a large temperature difference (i.e. flame retarding action) following a relatively flat potential field. (Because, self-restriction of landscape and inhomogeneity of material properties shall be negligible in active layer). So in turn, for ROI, the walker would move to adjacent sites with a more or less equal probability, converging to a RW algorithm similar to one above.

#### Self-expanding system

For silicone-based char geometry, the random distribution of worm-like clusters were generated by using a geometrical site percolation^61^ algorithm. It was simulated on a triangular lattice; each site is represented by hexagonal element as in Fig. 8c (not to be confused with honeycomb lattice having different critical threshold value^43^). The choice of lattice type was demonstrated by the intersection of site percolation at critical limit with fractal clusters^43,44^. The lattice points were independently defined with probability *p* of being open (only neighboring sites cluster, there is no special correlation between different sites^43^). The probability *p* was estimated from the void fraction of CT slices (Fig. 7c) using ImageJ. Distance between lattice nodes is *a*_*u*_ = *a*_*u*,*S*_.

### Software

Software versions used in this paper were: gcc6.4.0 libraries and MatlabR2016a^58^ for numerical simulations and analyses, MatlabR2016a^58^ for post-processing and data plots, ImageJ1.50i^62^ and its relevant plugins for image and CT volume treatments, UCSF Chimera1.10.2^63^ and ImageJ1.50i^62^ for 3D tomography plots and videos, Inkscape0.91 for schematic illustrations and the preparation of figures.

## Supplementary information

Video 1

Video 2

Video 3

Video 4

Video 5

Video 6

Supplementary information (text + figure)
