# Supplementary material for: Fractal conceptualization of intumescent fire barriers, toward simulations of virtual morphologies
Source: Sci Rep. 2019 Feb 12;9:1872. doi: 10.1038/s41598-019-38515-9 (PMC6372717; doi:10.1038/s41598-019-38515-9)
Supplement: Supplementary file 7 — Supplementary information (text + figure) [file 41598_2019_38515_MOESM7_ESM.docx]

**Supplementary information**

**Fractal conceptualization of intumescent fire barriers, toward simulations of virtual morphologies**

**Gizem Okyay, Anil D. Naik, Fabienne Samyn, Maude Jimenez & Serge Bourbigot***

Univ.Lille, CNRS, ENSCL, UMR 8207, UMET, Unité Matériaux et Transformations, F-59000 Lille, France

*Correspondance: serge.bourbigot@ensc-lille.fr

**ABSTRACT**

This document provides supplementary figures with details on the methodology used. The supplementary data is not crucial to understand and reproduce the work of the main text. However, it might be useful, especially for non-specialists of each technique used (fire testing, intumescence, image analyses), for a better understanding of the specimens used by means of their extended visualizations. There are five brief sections:

1. Sampling of the expanded coatings after fire testing

2. Uncensored data from electron microprobe analyzer

3. Uncensored data for pore observations on CT slices

4. Self-similarity checks on CT slices

5. List of supplementary videos

6. Notes on active walk model (AWM)

**1. Sampling of the expanded coatings after fire testing**

Intumescent coatings/paintings are used to protect materials against heat in case of fire hazard. Those coatings of few millimeters expand up to few centimeters: samples expanded on 10x10x0.3cm^2^ steel plates are shown in Fig. S1 (corresponding to the sketch of Fig. 1.a in the main text). The kinetics are complex and, generally, the best coating formulations are determined in an iterative tuning of kitchen recipes through small scale fire testing (Fig.S1.a). In order to obtain even better formulations, it is necessary to understand the modes of actions in detail: therefore, the morphology becomes an important parameter resulting from the modes of action and affecting the physical processes. Therefore, we selected two different modes of action of best formulations (reported among formulations with best performance).


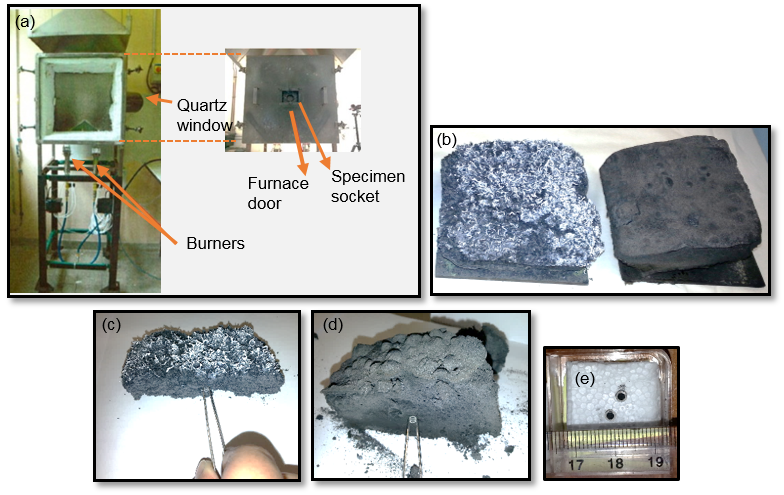


Supplementary Figure S1. (a) Bench scale furnace developed in our laboratory to simulate fire including ASTM119/ISO834 and UL1709 curves. (b) Expanded coatings on 10x10 cm^2^ steel plates after the bench scale hydrocarbon fire testing. Two types of formulations were tested: formulation with silicone binder (left) and epoxy binder (right). The inner structure of those intact samples were computerized by X-ray computed microtomography (CT). (c,d) Sample cuts for visual observations of the inner morphology and the collection of tiny specimens for higher resolution tomography (HRCT). (e) Tiny specimens were collected inside small plastic tubes transparent to X-rays. Numbered ticks of the ruler are on cm scale. For HRCT samples, only 1 mm^3^ were analyzed locally in the core, in order to minimize the errors which may arise from sample damage due to cutting. Note that the specimens were very crumbly as depicted in (c) and no experimental protocol exists at present on the cutting of intumescent samples without any structural damage.

**2. Uncensored data from electron probe microanalyzer**

The uncensored data is provided for EPMA (electron probe microanalyzer) recordings. The data is presented for epoxy char in Fig. S2 and for silicone char in Fig. S3 (corresponding to cut samples presented in Fig.2.d and Fig. 3.d of the main text respectively).


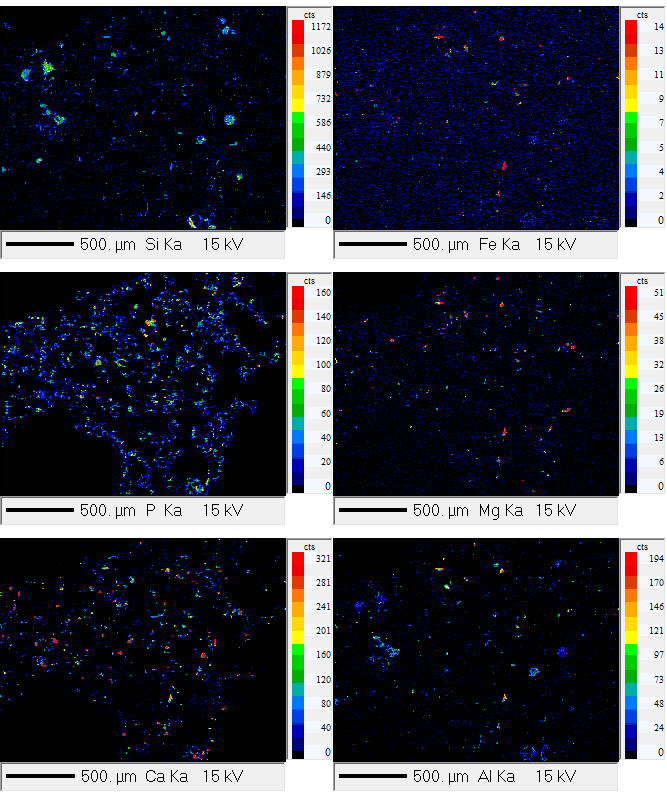

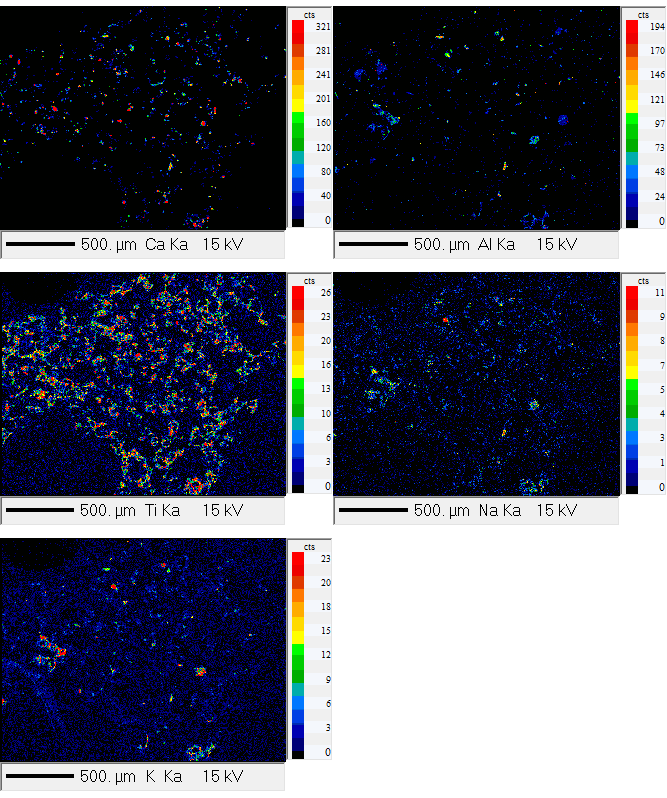

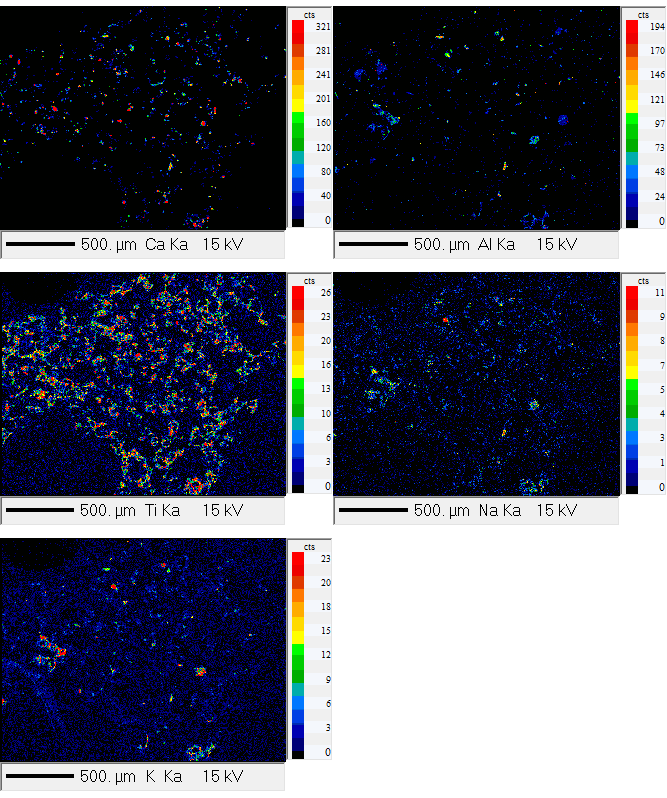


Supplementary Figure S2. Chemical mapping of selected region on epoxy based sample.


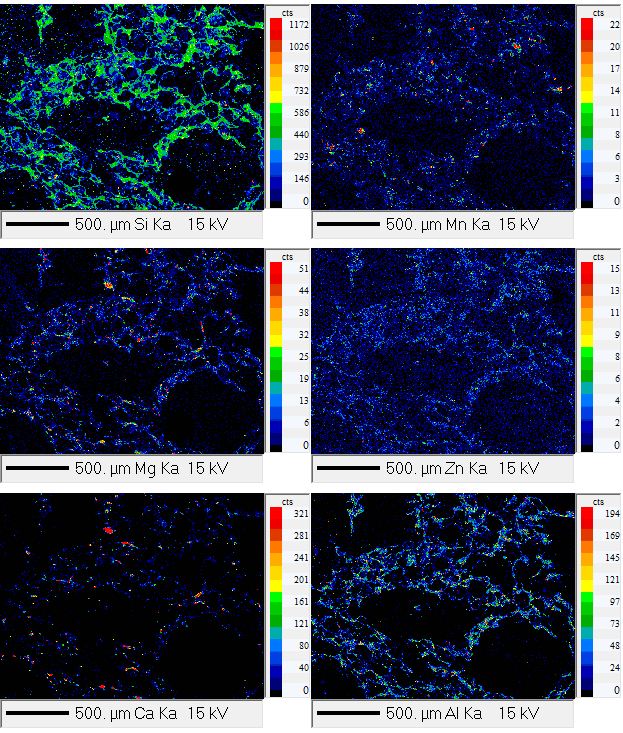

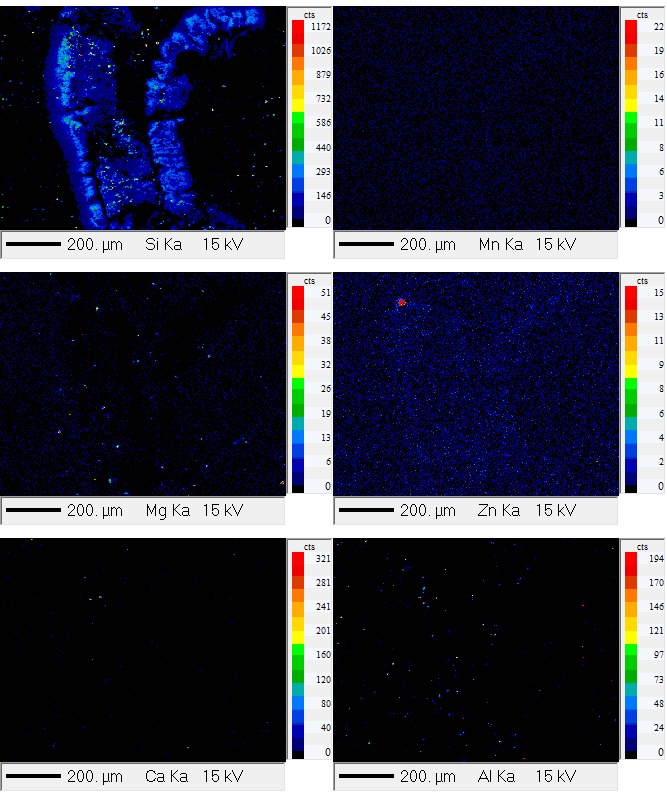


Supplementary Figure S3. Chemical mapping of selected regions on silicone based sample.

**3. Uncensored data for pore observations on CT slices**

The extended figures in order to visualize the separation of pores are presented in Fig. S4 (epoxy sample) and in Fig. S5 (silicone sample), corresponding to Fig. 2 and Fig. 3 of the main text respectively.


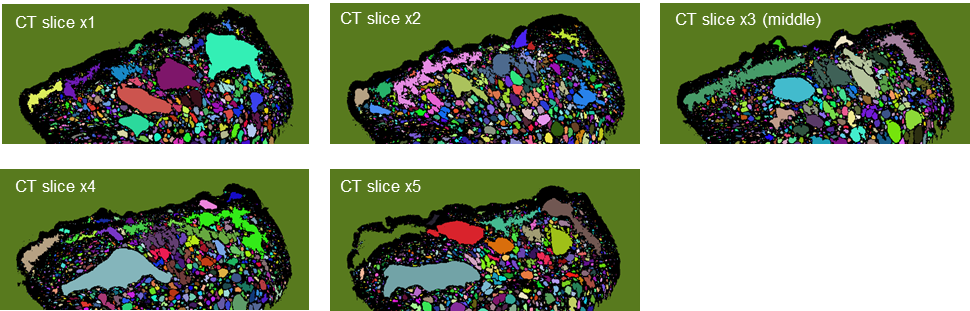


Supplementary Figure S4. The connectivity of void spaces for epoxy char, visualized after applying graylevel watershed on the image stack of five CT slices. It is noted that different sizes of bubbles/voids are randomly distributed. The interconnectivity seems to be higher at the very top layer of the char. This connection is due to higher temperatures in this area during fire tests, both leading to further accumulation of gases, and to some pyrolysis, leading to the breakage of walls followed by the further expansion of the active bottom layer.


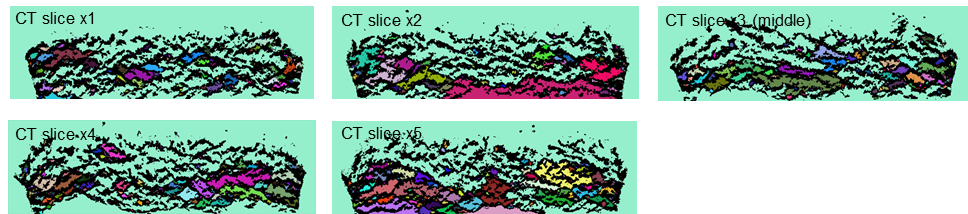


Supplementary Figure S5. The connectivity of void spaces for silicone char, visualized after applying graylevel watershed on the image stack of five CT slices. It is noted that the horizontal solid layers are quite uniformly separated. The interconnectivity is higher than the epoxy char. Both this uniformity and the interconnectivity is due to the mode of action based on self-expansion of EG (expandable graphite) platelets bonded by silicone based binder.

**4. Self-similarity checks on CT slices**

The self-similarity checks of fractal analyses are presented in Fig. S4 (epoxy sample) and in Fig. S5 (silicone sample) respectively, corresponding to the selected gray level threshold value (Fig.4.a of the main text). Each section is composed of 5 slices (*x1,x2,x3,x4,x5* in the main text.)


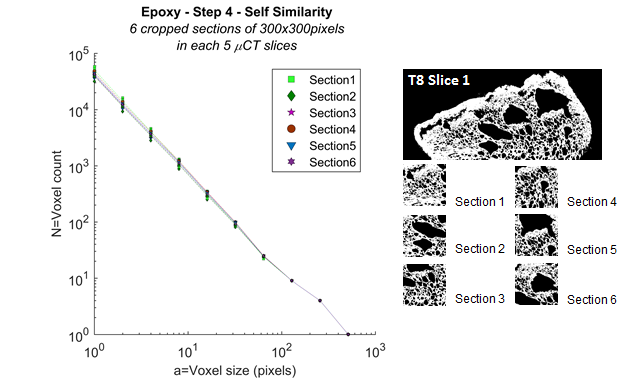

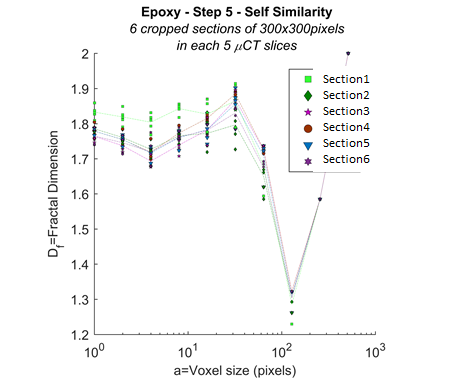


Supplementary Figure S6. Self-similarity check of the epoxy char on CT slices.


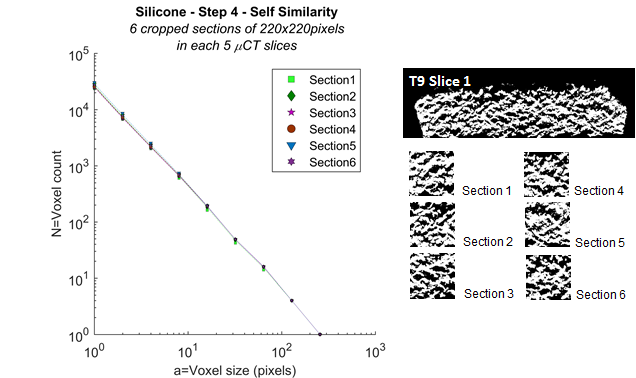

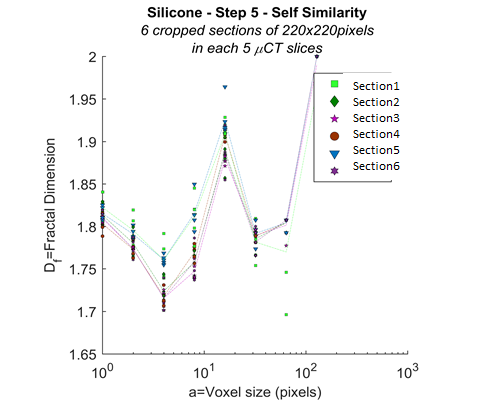


Supplementary Figure S7. Self-similarity check of the silicone char on CT slices.

**5. List of supplementary videos**

In the main text, the slice images were analyzed because the intumescence was conceptualized as a 2D problem. Nevertheless, for a better visualization of the problem, some 3D reconstructions of the specimens are provided in the form of videos:

- **Supplementary Video 1.** Rotation movie of **epoxy** based sample after **CT** reconstruction (bounding box of approximately **10x10x4 cm^3^**, tomography resolution of **81 µm/pixel**). Note the noisy signals on the sample corners: it is due to scattered X-rays during acquisition by the steel plate adhered to specimen.
- **Supplementary Video 2.** Rotation movie of **silicone** based sample after **CT** reconstruction (bounding box of approximately **10x10x2.5 cm^3^**, tomography resolution of **81 µm/pixel**). It is less noisy compared to HRCT-epoxy, because the steel plate was detached easily prior to acquisition.
- **Supplementary Video 3**. Rotation movie of **epoxy** based sample after **HRCT** reconstruction (bounding box of approximately **1 mm^3^**, tomography resolution of **1 µm/pixel**). Note the reconstruction noise on the corners due to ring artifacts and rotation axis errors.
- **Supplementary Video 4**. Rotation movie of **silicone** based sample after **HRCT** reconstruction (bounding box of approximately **1 mm^3^**, tomography resolution of **1 µm/pixel**). It is less noisy compared to HRCT-epoxy, because the elements are heavier causing higher absorption contrast with air.
- **Supplementary Video 5**. Rotation movie of the filtered pores (with size ~ **a_u_**) from **CT** reconstruction of **epoxy** based sample.
- **Supplementary Video 6**. Rotation movie of the filtered pores from **CT** reconstruction of **epoxy** based sample. Only the pores with **circularity C>0.8** are visualized: they are assumed to be “fresh bubbles” indicating the most active regions inside the specimen (i.e. regions with continuous inception and expansion) during fire testing. Their size correspond to around **a_u_** . Circularity (C=4*π *area/perimeter^2^) was computed in ImageJ from binarized images of CT slices at selected threshold level (Fig.4.a for the main text).

**6. Notes on active walk model (AWM)**

In this study, epoxy-based specimen was chosen for a preliminary demonstration of AWM for intumescence. In this specimen, the developed (“old”) layers has a non-uniform aspect, which necessitates the use of a more sophisticated model (other than random walk, aggregation-like or percolation). This is due to the chemical mode of action of the bubbling intumescence and charring, changing over time, so, as a function of expansion ratio. In AWM model, we need to define three items [Lam (2005)]: the *landscaping/potential function* dependent on the walker, the *stepping rule* of the walker and the *self-evolution of the landscape* independent of the walker. For fire retarding intumescence, the most straightforward item shall be the second one: the walker moves upon heat excitation (i.e. temperature in specimen). So, the stepping rule of the walker can formulated much like in BAW (Boltzmann Active Walk), where the probability of movement to adjacent sites can be parametrized using temperature variable [Lam (2005)].

We know apriori that our specimens expand under certain thermal gradient. The active intumescence (unconstrained bubbling action leading to heat retardation) occur under the most steep $\Delta T$. This is the temperature difference between the top side and the bottom side of an initial building block, before and during the expansion of this block. This difference eventually decays with time after first two steps of expansion, i.e. bubble growth and expansion, until coalescence. This action was illustrated in Figure 6.c of the main text (random movement and expansion of seeds, before complete coalescence). After those steps, there are no more active seeds in this building block: the block migrates away toward charring crust and the heat is already diffused toward subsequent layers, so, $\Delta T$decreases for that block. Thus, the walkers are restrained and the probability of movement (of each walker, i.e. each seed) shall be formulated similar to BAW:

$P_{ij}\propto exp\{ \beta[V(i)-V(j)] \}$ (S1)

where the proportionality constant is $\beta\propto1/\Delta T$;$V$ is the landscape potential (or vector potential) modified by the motions of walkers. (Lam (2005)).

Here the difficulty lies in the definition of the landscape because the governing parameter in fire retarding intumescence is unknown. Also, same specimen can exhibit different actions (not limited to active layer) under different excitations as convective heat, radiative heat, mechanical, etc. changing the reaction type, thus the morphology. As mentioned by Lam (2005) and previously by Kayser et al. (1992), there are numerous possibilities for the definition of the landscaping function; this is especially true for our intumescent samples involving three phase material with many simultaneous and transient parameters (non-exhaustive examples are: temperature, pressure, viscosity, conductivity, homogeneity of additives, type of additives for mechanical strength, processing of virgin coating [for initial background landscape], etc. ). So, we assigned a flat potential background, with a hypothetical step-wise landscaping rule (walker dependent and self-evolving combined) as illustrated in Supplementary Figure 8, with a certain conservation of W (landscaping rule, Lam (2005)) over slices $x_{1}$ to $x_{5}$. As depicted here, walkers in mid-bottom regions (middle in both y-axis and x-axis) (close to the most active intumescence, in the fully developed region) shall be less constrained with minimal effect on their active landscape. When they start to move upwards, they squeeze the above layers, hence they change the landscape, so the probability of moving upwards decays. Similarly, toward sides of the block, there is some blockage of movement proportional to the distance to y* (middle of specimen), and to the previous movements of the walker in the same region. Walking tracks are allowed to overlap; self-crossing is allowed. Walkers never die and the walking stops when the mass density (here, area density) of material is satisfied.


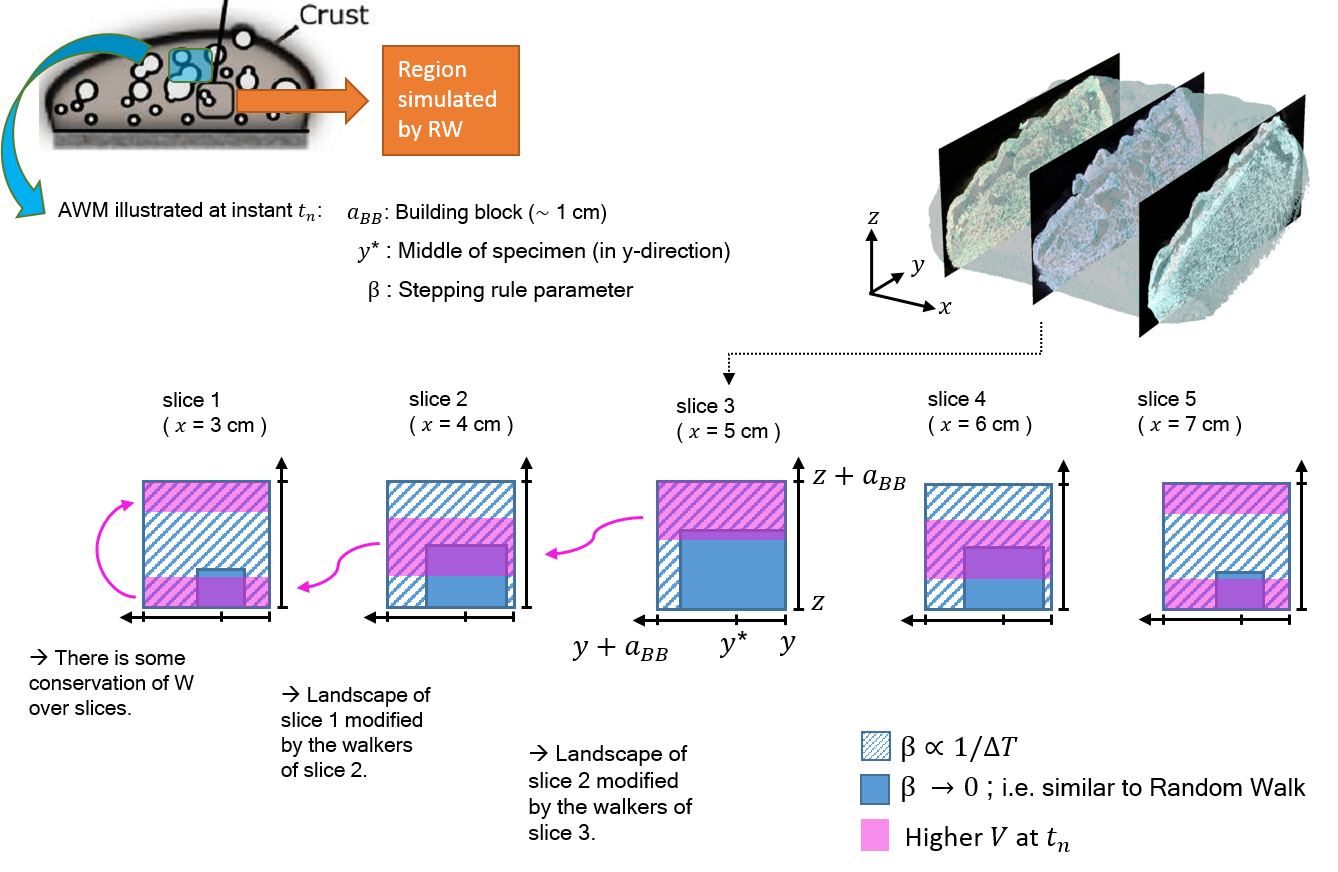


Supplementary Figure 8. Example of AWM for building blocks in upper char layer (i.e. near the top crust ≠ active layer).

Finally, note that at an instant t, all those above constraints will diminish for active layer with $\Delta T$ being maximum, so the method will eventually converge to a RW-like method giving blocks similar to the ones presented in the main text for the ROI of epoxy-based char. A more sophisticated model can be developed in future, when one has realistic data about the potential fields (temperature, pressure, etc.) inside the specimen.
